# Tailoring the chiral magnetic interaction between two individual atoms

**DOI:** 10.1038/ncomms10620

**Published:** 2016-02-23

**Authors:** A. A. Khajetoorians, M. Steinbrecher, M. Ternes, M. Bouhassoune, M. dos Santos Dias, S. Lounis, J. Wiebe, R. Wiesendanger

**Affiliations:** 1 Department of Physics, Hamburg University, 20355 Hamburg, Germany; 2 Institute for Molecules and Materials, Radboud University, 6525AJ Nijmegen, The Netherlands; 3 Max Planck Institute for Solid State Research, 70569 Stuttgart, Germany; 4 Peter Grünberg Institut and Institute for Advanced Simulation, Forschungszentrum Jülich & JARA, 52425 Jülich, Germany

## Abstract

Chiral magnets are a promising route towards dense magnetic storage technology due to their inherent nano-scale dimensions and energy efficient properties. Engineering chiral magnets requires atomic-level control of the magnetic exchange interactions, including the Dzyaloshinskii–Moriya interaction, which defines a rotational sense for the magnetization of two coupled magnetic moments. Here we show that the indirect conduction electron-mediated Dzyaloshinskii–Moriya interaction between two individual magnetic atoms on a metallic surface can be manipulated by changing the interatomic distance with the tip of a scanning tunnelling microscope. We quantify this interaction by comparing our measurements to a quantum magnetic model and *ab-initio* calculations yielding a map of the chiral ground states of pairs of atoms depending on the interatomic separation. The map enables tailoring the chirality of the magnetization in dilute atomic-scale magnets.

## Abstract (web summary)

The Dzyaloshinskii-Moriya exchange interaction arises in magnetic systems with broken inversion symmetry and promotes chiral magnetic order which may be exploited in spintronic devices. Here, the authors demonstrate how such an interaction between magnetic atoms on a metallic surface may be tuned by their separation.

## Graphical abstract

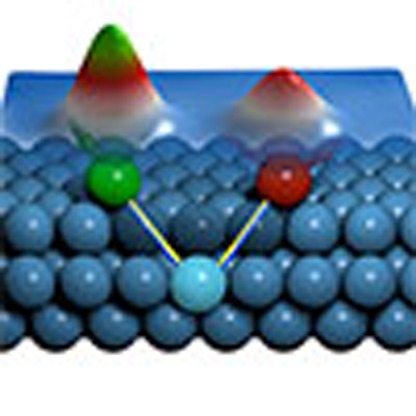

Chirality is a peculiar lack of symmetry, which is seeing an increasing importance in various fields of science from the structure of molecules^1^ to the magnetic properties of solids^2^. In the field of magnetism, chirality defines a rotational sense for the magnetization, which can rotate clockwise or counterclockwise along an axis of the magnetic object. Such chiral magnets have been recently shown to host many interesting properties that rely on this local rotation, or twisting, of the magnetization such as inducing new quantum information particles based on Majorana fermions^3^,^4^. So-called skyrmions^5^,^6^, a particular class of chiral magnetization structure, are attractive candidates for information technology as they are inherently nano-sized, and they respond more efficiently to spin-polarized currents^7^ as compared with conventional domain walls^2^,^8^,^9^. This energy-efficient aspect of chiral magnets, combined with their miniature dimensions, makes them technologically appealing as they can be integrated into current designs for high-density storage^2^,^10^. And yet, creating chiral magnets with a well-defined size or designed spin texture is a difficult task^11^,^12^ that has not been achieved yet in an atom-by-atom manner.

Central to the understanding of how chiral order emerges at the atomic scale is the Dzyaloshinskii–Moriya interaction (DMI)^13^,^14^, which is defined as *E*_DMI_=**D** ˙ (**S**_1_ × **S**_2_) (Fig. 1). The DMI is a fundamental ingredient responsible for twisting the magnetization of two coupled spins **S**_1_ and **S**_2_, as the overall energy can be reduced between the two spins by canting their relative orientation as dictated by the Dzyaloshinskii–Moriya (DM) vector **D**. Moriya proposed that the origin of this anisotropic magnetic exchange interaction can be derived by considering spin-orbit coupling within a superexchange model^14^. Later, Smith^15^ and Fert^16^ derived that DMI between **S**_1_ and **S**_2_ can also stem from an indirect exchange mechanism mediated by conduction electrons. Here, similar to the usual Ruderman–Kittel–Kasuya–Yosida (RKKY) interaction^17^, conduction electrons locally exchange-couple to the atomic spins becoming spin-polarized and thereby mediate an interaction. However, in addition, the electrons spin-orbit scatter at the non-magnetic host atoms (Fig. 1)^15^,^16^. This process leads to a long-range DMI between the atomic spins, where not only the amplitude, but also the orientation of the DM vector oscillates as a function of separation. The magnetization of the coupled pair of quantum spins with spin operators 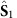 and 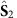 can then be quantified by the following Hamiltonian:


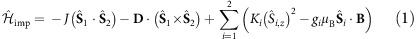


It decomposes into an isotropic Heisenberg exchange interaction term (*J*), which takes into account the usual RKKY-like exchange that favours collinear orientations, a DMI term (**D**), which favours non-collinear spin orientations, a single spin magnetic anisotropy term (*K*_*i*_), which locks each spin in a given orientation resulting from the interaction with the crystal field, and a Zeeman term, which considers the response of each spin to a magnetic field **B** as determined by the *g*-factors *g*_*i*_ and the Bohr magneton *μ*_B_. As the orientation of the DM vector in equation (1) dictates a sense of rotation of **S**_2_ compared with **S**_1_, the distance-dependent oscillation of **D** induced by the indirect exchange mechanism inherently contains the possibility to tune the chirality by the separation of the pair. Nevertheless, although the DMI interaction has been studied to a large extent for bulk^5^ or thin-film systems^9^,^18^, little is known experimentally about how the indirect conduction electron-mediated DMI emerges between individual atoms, as originally proposed by Smith^15^,^16^. Central to this understanding are methods that can interrogate exchange interactions^19^ with ultimate spatial resolution on surfaces^17^.

To this end, we probe the magnetic excitations of coupled pairs of single iron atoms on a Pt(111) surface by low-temperature inelastic scanning tunnelling spectroscopy (ISTS; see Methods section for details). The choice of Pt as a substrate is motivated by a large spin-orbit coupling^20^. It induces a considerable distance dependent, oscillatory DM interaction within the pair, which is of similar strength as the isotropic exchange over a wide range of iron atom distances in this system. We finally show how the chirality of the resulting highly non-collinear magnetization states of the pair can be tuned by tailoring the positions of the two atoms.

## Results

### Spectroscopy of the uncoupled constituents outside of the pair

To minimize the effect of magnetic anisotropy, which is typically strong for atoms on surfaces^20^,^21^, we use a clean Fe atom and an Fe atom with two attached hydrogen atoms (FeH_2_), each with spin *S*=5/2, which exhibit a very weak magnetic anisotropy depending on their adsorption site, face-centered cubic (fcc) or hexagonal close-packed (hcp), on the substrate atomic lattice^22^,^23^. At zero magnetic field *B*_*z*_, ISTS on Fe_hcp_H_2_ reveals a strong peak at zero bias characteristic for the Kondo effect^23^,^24^, whereas the bare Fe atom shows a symmetric step-like increase in conductance because of spin excitations of the magnetic ground state to the first excited state (Fig. 2a, grey curves). This zero-field splitting originates from the magnetic anisotropy. For Fe_hcp_, we found *K*=0.08 meV, and for Fe_fcc_, *K*=−0.19 meV (ref. 22). Owing to the small anisotropy in Fe_hcp_H_2_ (*K*=0.02 meV), at zero field all eigenstates are nearly degenerate^23^ at our base temperature and the Kondo impurity behaves paramagnetically^25^, making it extremely sensitive to the magnetic perturbations produced by *J* and **D**.

### Distance-dependent splitting of the Kondo resonance

By changing the interatomic distance in artificially arranged pairs of atoms with the tip of the scanning tunnelling microscope (STM)^26^ (see Methods section and Supplementary Fig. 1 for details), we demonstrate that the magnetic excitations of each spin are modified by the indirect exchange interaction depending on the interaction strength (Fig. 2a,b). We create different pairs, by leaving the Fe_hcp_H_2_ stationary and moving the Fe atom to a chosen distance from the centre of the Fe_hcp_H_2_ impurity (Fig. 2b). ISTS on each individual atom of a fabricated pair (Fig. 2a), reveals a splitting of the Kondo resonance on Fe_hcp_H_2_ (ref. 27) with a correlated increase in energy of the spin excitation on Fe as compared with the isolated impurities (see grey spectra in the foreground), which indicates the magnetic coupling between the two impurities. To quantify this interaction to first approximation, the splitting of the Kondo resonance at zero magnetic field can be mimicked by the effect of an effective exchange field |*B*_eff_| (Fig. 2c; see Methods section for details)^23^. For this purpose, the Kondo spectra are fitted to a Kondo model^25^,^28^, which considers the weak coupling limit within perturbation theory (see Methods section for details). The resulting |*B*_eff_| reveals a strong distance-dependent oscillation illustrating the long-range nature of the magnetic coupling reminiscent of the RKKY interaction^17^,^29^. As the separation of the magnetic atoms in the pairs are too large for a significant contribution of direct or super-exchange, the behaviour in |*B*_eff_| strongly suggests that the observed coupling is due to the indirect substrate electron-mediated exchange mechanism.

### Determination of Heisenberg and DM exchange

**Figure 1:**
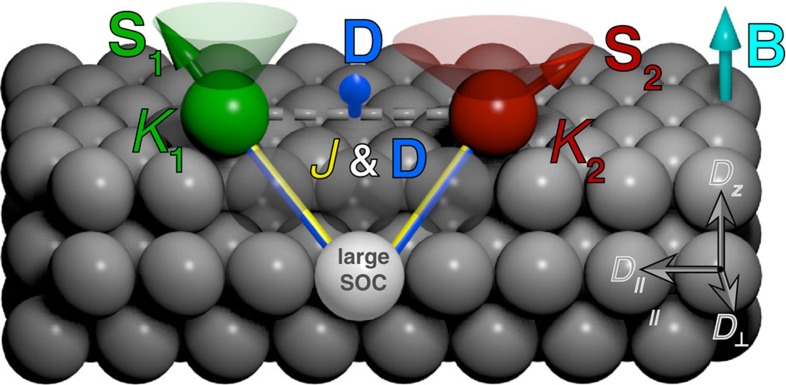
Cartoon diagram of the indirect conduction electron-mediated DM interaction. The interaction between two magnetic atoms (red/green) is mediated by scattering of conduction electrons at a substrate atom (grey) with strong spin-orbit coupling (SOC)^2^,^15^,^16^. The shown scattering process leads to a contribution to the Heisenberg-like exchange (*J*) and DM vector (**D**), which is oriented perpendicular to the indicated triangle constituted by the two magnetic atoms and the substrate atom. The overall *J* and **D** are given by the contributions of all substrate atoms resulting in a nonzero **D**=(*D*_||_, *D*_⊥_, *D*_*z*_) (orientation of the components as indicated) because of broken inversion symmetry at the surface^18^. The orientation of the spins **S**_1_ and **S**_2_ of the coupled pair is determined by the interplay between the single ion magnetic anisotropy of each atom (*K*_*i*_), *J*, **D**, and the applied magnetic field (*B*_*z*_).

**Figure 2:**
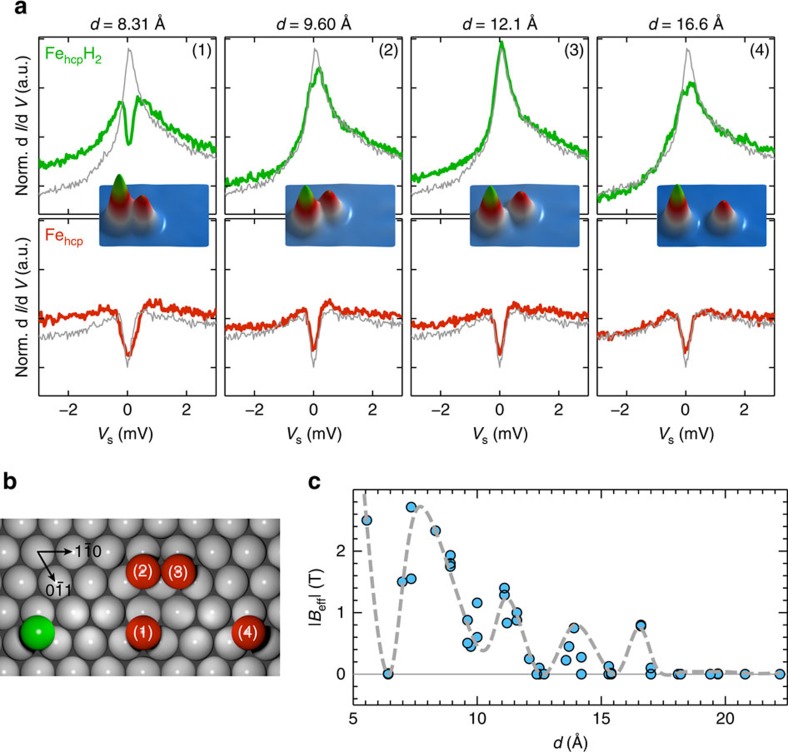
Detection of the indirect conduction electron-mediated exchange interactions. (**a**) ISTS on Fe_hcp_H_2_ (green) and Fe_hcp_ (red) within selected pairs of different configuration (**b**) and separation *d* (topographs shown in insets) in comparison to the spectra measured on the corresponding isolated atoms (grey). The exchange interaction within the pair forces a splitting of the Kondo resonance of Fe_hcp_H_2_ and a modification of the magnetic excitation of Fe as compared with the isolated atoms (experimental ISTS parameters: *V*_s_=6 mV, *I*_s_=3 nA, *V*_mod_=40 μV, the spectra are normalized (Norm.) by division by a substrate spectrum measured with the same tip). (**b**) Positions of Fe_hcp_H_2_ (green) and Fe_hcp_ (red, 1–4) for the pairs of **a** with respect to the substrate atoms (grey). (**c**) The exchange field resulting from magnetic coupling is mimicked as an effective magnetic field (|*B*_eff_|), which is determined by fitting the splitting of the Kondo resonance of pairs of different distances *d*. The grey dashed line is a guide to the eye and the maximum error bar was determined to be 0.5 T.

**Figure 3:**
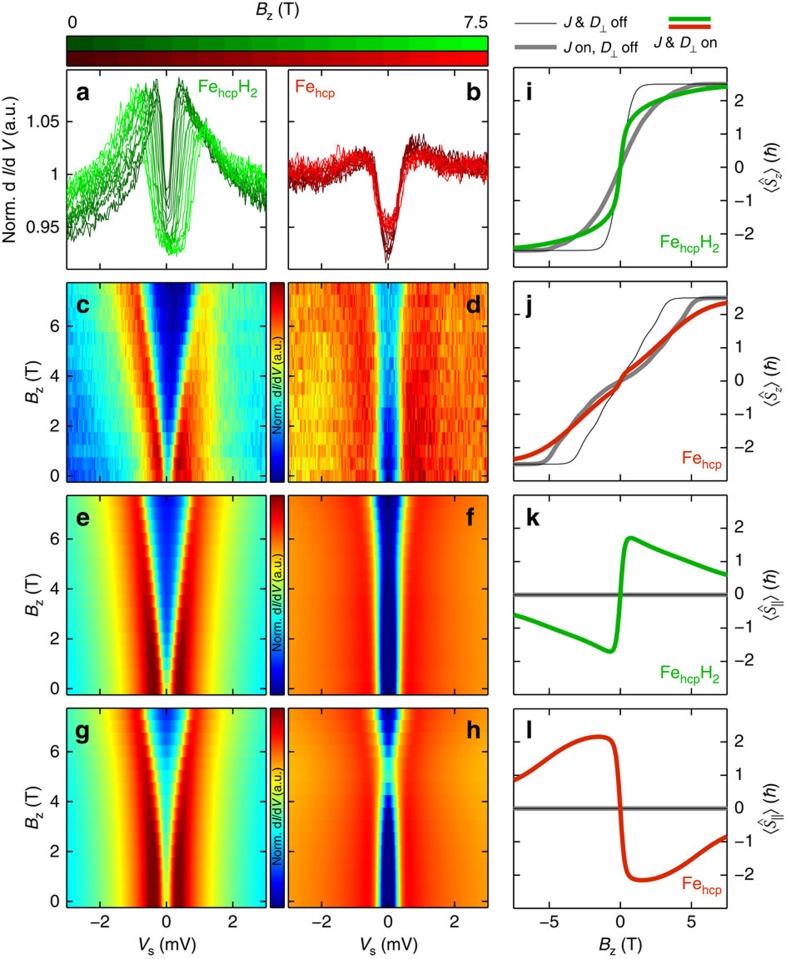
B-field dependence of spin-excitation and magnetization of a DM-coupled pair. (**a**,**b**) ISTS measured on Fe_hcp_H_2_ and Fe_hcp_ within a pair of separation *d*=8.31 Å as a function of magnetic field as indicated at the top. (**c**,**d**) The same data as a colour intensity plot where the colour indicates the 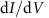 signal (experimental ISTS parameters: *V*_s_=6 mV, *I*_s_=3 nA, *V*_mod_=40 μV, the spectra are normalized (Norm.) by division by a substrate spectrum measured with the same tip). (**e**–**h**) Simulation of the spectra using a Kondo model that takes into account the exchange interaction within the pair including (**e**,**f**; 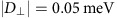, *J*=−0.06 meV) and excluding ((**g**,**h**) 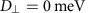, *J*=−0.06 meV) the DMI. (**i**–**l**) Simulated expectation values of the out-of-plane (**i**,**j**) and parallel (**k**,**l**) spin components of both atoms in the pair as a function of magnetic field for various settings of *J* and |*D*_⊥_| as indicated. The parameters within all plots of this figure are 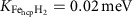, 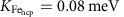, 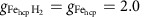.

To disentangle the contributions of *J* and **D** to the observed indirect exchange interaction (Fig. 1)^15^, we monitor the simultaneous changes in both the Kondo resonance of Fe_hcp_H_2_ and the magnetic excitation of Fe as a function of an applied out-of-plane magnetic field (*B*_*z*_). This is shown in Fig. 3a–d for a particular pair with *d*=8.31 Å. The corresponding data for pairs with other distances are described in Supplementary Note 1. With increasing *B*_*z*_ the Kondo resonance splits nearly linearly indicating that the impurity easily aligns to the magnetic field direction, with only a small deviation compared with an isolated Fe_hcp_H_2_ (ref. 23). However, Fe_hcp_ shows a *B*_*z*_-dependent evolution, which largely deviates from the isolated impurity^22^. The magnetic excitation shows practically no shift up to *B*_*z*_=6 T and does shift linearly to higher energies only for larger magnetic fields. For the isolated Fe_hcp_ atom, 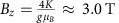 is required to overcome the easy-plane anisotropy and align the spin fully parallel to the magnetic field direction (that is, into the *S*_*z*_=5/2 state). In the magnetic field-dependent map of the spin excitation of the isolated Fe_hcp_ atom^22^, this is manifested by a minimum in the excitation energy at *B*_*z*_=3.0 T, which is induced by the level crossing of the *S*_*z*_=5/2 and *S*_*z*_=3/2 states, followed by a linear shift in the excitation at higher magnetic fields. The absence of this minimum for the Fe_hcp_ within the pair (Fig. 3d) signifies a strong magnetic coupling, which destroys the level crossing.

To understand how *J* and **D** separately affect the spectra, we simulate the measured ISTS of each atom considering the Hamiltonian equation (1) within the Kondo model (see Methods section for details)^28^ for different values of *J* and **D** and fixed values of *K* and *g*, which have been determined by fitting the isolated atoms (see Methods section for details). By considering the simplified superposition of the contributions of all substrate atoms to **D** shown in Fig. 1 we expect the out-of-plane component *D*_*z*_ as well as the in-plane component *D*_||_, which is parallel to the displacement vector between the two atoms, to be negligible for most of the pairs, whereas only the in-plane component *D*_⊥_ pointing perpendicular to the displacement vector survives due to the broken inversion symmetry induced by the surface. This is later confirmed by our first-principles calculations (see below). To reduce the number of parameters, we therefore set *D*_*z*_=*D*_*||*_=0 and only consider the absolute value of *D*_⊥_. The latter restriction is furthermore justified as equation (1) does not contain any operators besides **D** that break the in-plane symmetry. Figure 3e,f exemplarily shows the simulated spectra for the *d*=8.31 Å pair that best fit the experimental ISTS (Fig. 3c,d) as determined by a variation of both, *J* and |*D*_⊥_|, whereas the same is shown in Fig. 3g,h but with the restriction of zero DMI (*D*_⊥_=0). Obviously, only when considering the combined effect of nonzero **D** and *J*, is it possible to excellently reproduce the trends in the experimental spectra for both Fe_hcp_H_2_ and Fe_hcp_. In particular, the effect of nonzero **D** is to avoid the level crossings of the Fe_hcp_ spin states resulting in the experimentally observed absence of the above-mentioned minimum in the excitation energy. Moreover, the almost linear splitting of the Fe_hcp_H_2_ Kondo resonance, which behaves very similar to the splitting for the isolated impurity^23^, is nicely reproduced. The influence of the DMI on the magnetization is illustrated in Fig. 3i–l by the magnetic field-dependent expectation values of the spin in out-of-plane 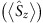 and parallel 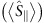 orientations (see Methods section for details). The most dramatic effect of **D** is the appearance of strong parallel components of the magnetization on both atoms in the pair (Fig. 3k,l). The parallel component is positive for Fe_hcp_H_2_ and negative for Fe_hcp_ at positive *B*_*z*_, and vice versa for negative *B*_*z*_, illustrating the creation of non-collinear, antiferromagnetic magnetization states. Moreover, the difference in the sign of the parallel components defines a rotational sense of the magnetization when going from Fe_hcp_H_2_ to Fe_hcp_, demonstrating the chiral nature of the DMI.

### Distance dependency of Dzyaloshinskii–Moriya exchange

**Figure 4:**
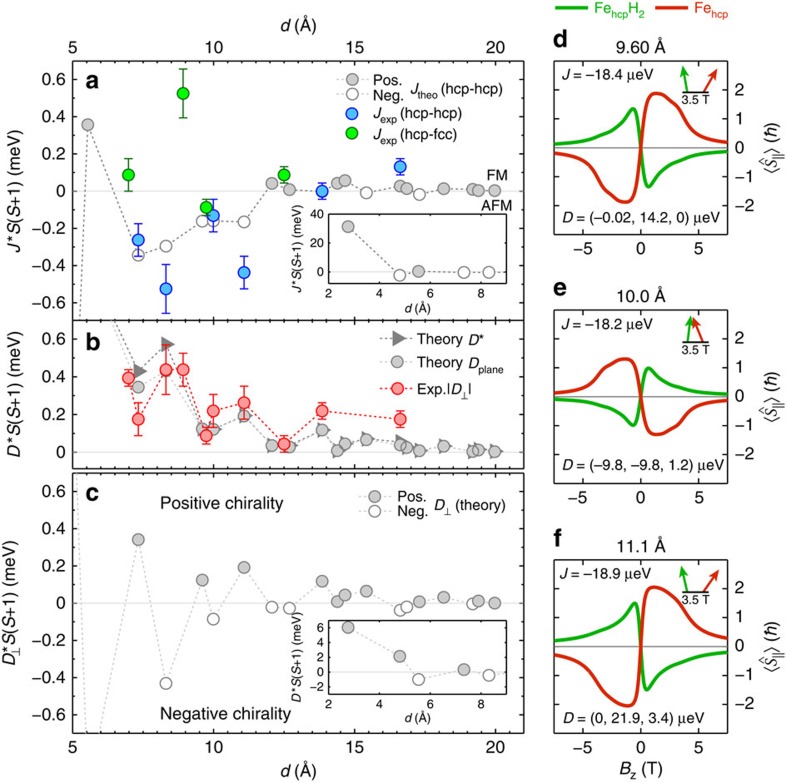
Distance dependence of indirect conduction electron-mediated DM interaction. (**a**–**c**) Experimentally determined values of *J* and |*D*_⊥_| (coloured circles, error bars are estimated by the largest variation of the two parameters, which still results in a reasonable fit to the data) compared with the *ab-initio* calculation of *J*, *D*_⊥_, 
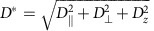
 and 
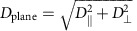
 using the KKR approach (grey and white circles and triangles). The values are scaled by 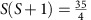 for direct comparison to previous publications on related systems^17^,^29^. The theoretical data are calculated for a pair of two clean Fe atoms and technically restricted to hcp–hcp pairs only. Insets illustrate theoretical data at small separations. The dashed lines are made to guide the eye. (**d**–**f**) Simulated magnetic field-dependent expectation values of the parallel spin components of both atoms in three selected pairs with negative *J* and **D**=(*D*_||_, *D*_⊥_, *D*_*z*_) as indicated, and 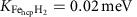, 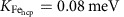, 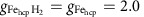. The arrows in the insets indicate the orientations of the magnetizations of Fe_hcp_H_2_ (green) and Fe_hcp_ (red) defined by the three components of the spin expectation values 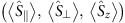. Neg.: negative; Pos.: positive.

It is interesting to investigate how the two indirect conduction electron-mediated exchange components **D** and *J* vary as a function of distance. It has been shown that the sign and amplitude of *J* is distance dependent, as determined by the electronic structure of the atom/substrate complex^17^. However, it is not *a priori* clear if the twisting and the chirality induced by **D** oscillates in a similar way^30^,^31^. To this extent, we vary the separation *d* between the atoms in the pair and perform a similar measurement and analysis as shown in Fig. 3 in order to extract *J* and |*D*_⊥_| for all pairs (Supplementary Note 1) within an errorbar whose estimation we explain in Supplementary Note 2. The resulting distance dependence shown in Fig. 4a,b reveals a damped oscillation in *J* between antiferromagnetic (*J*<0) and ferromagnetic (*J*>0) coupling, which reflects the well-known behaviour of indirect conduction electron-mediated Heisenberg exchange^17^. More importantly, we see that also |*D*_⊥_| shows a strong distance dependence, and long-range oscillations with an apparently smaller wavelength as compared with *J*, which we discuss below. This oscillatory behaviour of |*D*_⊥_| further substantiates that the origin for the observed DMI is indirect conduction electron-mediated exchange as proposed by Smith^15^. As we have shown for Co/Pt(111), this indirect exchange interaction between single atoms is intricately related to the electronic structure^17^, and first-principles calculations have shown reasonable agreement with the experimental values of *J*. To understand the distance-dependent evolution of *J* and **D**, we therefore employed first-principles calculations based on density functional theory as implemented in the full-potential Korringa–Kohn–Rostoker Green function (KKR) method (see Methods section for details)^32^, which includes spin-orbit coupling.

The theoretical results are summarized and compared with the experimental values in Fig. 4a–c, where we compare the experimentally extracted values of *J* and |*D*_⊥_| to calculations for Fe_hcp_-Fe_hcp_ pairs using a mapping to a classical Heisenberg model (see Methods section for details). The values of *J* shown in Fig. 4a are obtained after taking the trace of the tensor of exchange interactions for the two adatoms. We note that the variation between the different diagonal terms of the tensor is extremely small, justifying the use of an isotropic *J* in our model Hamiltonian. Moreover, although in principle also biquadratic exchange interaction can contribute to the Hamiltonian, this contribution turns out to be negligible when the distance between the magnetic atoms is large (see Methods section for details). By comparison of the length of **D** 
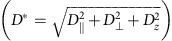
 to the in-plane component 
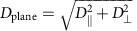
 and to *D*_⊥_ (Fig. 4b,c), we see theoretically that the *D*_⊥_ component is the dominant term compared with the other components of **D** for most of the pairs. Only at distances *d*<9 Å, small but significant values of *D*_*z*_ lead to a deviation between D* and *D*_⊥_ (Supplementary Note 3). This justifies the simplified determination of negligible components of **D** via the construction illustrated in Fig. 1 and the restriction to *D*_⊥_ used in the simulations of the experimental spectra. Most importantly, the calculated *D*_plane_ quantitatively reproduces the experimentally determined values of |*D*_⊥_|, in particular the apparently smaller wavelength of the oscillation of |*D*_⊥_| as compared with that of *J*. As the experimental determination is not sensitive to the sign of *D*_⊥_, the oscillations are more clearly revealed by the calculated values of *D*_⊥_ shown in Fig. 4c. These oscillations indeed show sign changes on a much shorter length scale as the oscillations in *J*. This is qualitatively expected already from the simple model where the interactions are mediated by a quasi-free electron gas^15^. Here, the long-range form of *J* is given by sin(2*k*_*F*_*d*), whereas that of **D** is given by sin(*k*_*F*_[*R*_*A*_+*R*_*B*_+*d*]), where *R*_*A*_ and *R*_*B*_ are the distances of the two atoms A and B of the pair to the substrate atom mediating the DMI (Fig. 1). As *R*_*A*_+*R*_*B*_+*d*≥2*d*, the DMI is expected to oscillate on a smaller length scale as the Heisenberg exchange. However, it should be noted that the realistic band structure of the substrate, which yields highly anisotropic energy contours in *k*-space, results in a more complex behaviour than expected from these simple models^17^.

## Discussion

The oscillation of the sign of the dominating DM component *D*_⊥_ illustrated in Fig. 4c directly implies that the orientation of **D** changes as a function of the separation of the pair, namely between pointing to the left or to the right looking along the displacement vector between the two atoms. As the DMI favours a particular rotational sense of the magnetization in the pair which depends on the orientation of **D** (equation (1)), the oscillations have a direct consequence on the preferred chirality of the magnetization in the pair. This is visualized in Fig. 4d–f, where we show the parallel components of the expectation values of the spins of three antiferromagnetic pairs with positive (d,f) and negative (e) *D*_⊥_. At positive *B*_*z*_, the sign in *D*_⊥_ leads to a negative/positive parallel component on Fe_hcp_H_2_/Fe_hcp_ for *D*_⊥_>0 and vice versa for *D*_⊥_<0. Taking into account the *z*-components of the expectation values of the spins, this implies a clockwise or counterclockwise rotation of the magnetization, respectively, when going from Fe_hcp_H_2_ to Fe_hcp_, as indicated by the magnetization vectors in the insets of Fig. 4d–f (see Supplementary Movie 1 for details).

The oscillation in the indirect conduction electron-mediated DM interaction proposed by Smith in 1976 (ref. 15), which we experimentally verify here, consequently enables tailored chirality of the magnetization in pairs of atoms by tuning the interatomic distance. More specifically, by changing the interatomic distance, the strength of the DMI can be of the same magnitude or even larger than that of the isotropic exchange interaction, which drives collinear order. The measured and calculated interaction maps (Fig. 4), combined with small magnetic anisotropy energies^23^ enables a class of bottom-up magnets with tunable chirality, for example, of a two-dimensional nature, such as magnetic skyrmions^9^, down to one-dimensional systems, such as spin spirals^33^.

## Methods

### Experimental equipment, tip and sample preparation

All experiments were performed with an STM in an ultrahigh vacuum facility at 0.3 K (ref. 34). A magnetic field *B*_*z*_ of up to 12 T can be applied perpendicular to the sample surface. Topography images were obtained in constant-current mode with stabilization current *I*_s_ and voltage *V*_s_ applied to the sample. The ISTS were obtained by using a lock-in technique to record the differential conductance 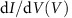 while ramping the bias voltage (*V*_S_) after the feedback loop was switched off, by adding an AC modulation voltage *V*_mod_ (given in rms) to the bias voltage.

We used a clean tungsten tip and a tungsten tip that was initially coated with nominally 50 monolayers of chromium before inserting it into the STM as described in ref. 34. For the ISTS shown here, both tips were afterwards prepared by voltage-pulsing and dipping into the platinum surface until almost featureless spectra on platinum and close to symmetric spectra on the magnetic atoms were achieved.

The Pt(111) single crystal was cleaned by alternating Ar^+^ sputtering (high voltage=2 kV) and annealing (≈740 °C) cycles of decreasing duration. An oxygen annealing procedure has been performed for 1 h at ≈660 °C and an O_2_ pressure in the chamber of 2 × 10^−6^ mbar. The final flash was carried out at ≈1000 °C for 1 min and a background pressure of 5.5 × 10^−10^ mbar.

Finally, Fe was deposited onto the clean Pt(111) surface. To obtain single atoms on the surface, the crystal was cooled to <10 K during deposition. This resulted in a statistical distribution of Fe atoms adsorbed to the fcc (Fe_fcc_) or hcp (Fe_hcp_) hollow sites of the Pt(111) surface, where the adsorption site can be identified by the characteristic ISTS spectra^22^. In addition, we recorded an atomic manipulation image in Supplementary Fig. 1a by recording a constant current image with an adatom partly bound to the tip^35^. This allows us to exactly determine the lattice of the Pt(111) surface and to align the direction of close packed Pt surface atoms parallel to the fast scan direction.

After long-term exposure of the sample to H_2_ from the residual gas of the ultrahigh vacuum chamber, some of the Fe atoms form Fe–H complexes with one or two attached H atoms. Using ISTS, the complex with two attached H atoms where the Fe is sitting on the hcp site (Fe_hcp_H_2_) can be identified by a characteristic Kondo peak in ISTS spectra^23^. Although clean Fe can be laterally manipulated between the two different hollow adsorption sites with typical manipulation parameters (*V*_s_=2 mV, *I*_s_=40–60 nA), it is not possible to manipulate Fe_hcp_H_2_ without de-attaching at least one H atom. Therefore, for the creation of the different pairs investigated here, Fe_hcp_H_2_ was kept stationary and Fe was moved to different positions. The Pt lattice determined by the atomic manipulation image together with the spectroscopic information from ISTS enables unambiguous determination of the absolute positions of the atoms in the pairs with respect to the Pt lattice underneath. Examples for two pairs with different distances, angles and adsorption sites are given in Supplementary Fig. 1b,c.

### Model for simulations of conductance spectra and magnetization curves

To extract the values for the Heisenberg coupling *J* and the DMI **D** from our experiments, we used a third-order perturbation theory model^25^,^28^ to fit and simulate our spectroscopic data. The Hamiltonian for a number *n* of impurities in this model consists of a Zeeman and an anisotropy term and looks like this:


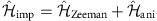



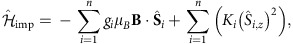


with *g*_*i*_ as the gyromagnetic factors, *μ*_B_ the Bohr magneton, **B** the applied magnetic field, 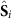 the vector of spin matrices 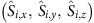 of the impurity and *K*_*i*_ the uniaxial anisotropy constant.

An additional coupling Hamiltonian considers the isotropic Heisenberg and the anisotropic DMI between all pairs of atoms. The interaction Hamiltonian then looks like the following:


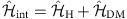



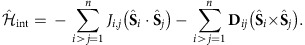


We calculate conductance spectra using a perturbative model where we take into account the scattering matrices up to the third-order using the following Kondo-like interaction matrix between the localized spin system and the electrons, which tunnel between tip and sample:


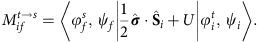


Here, 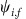 represent the initial and final wave functions of the probed spin, 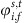 are the electron states in sample or tip, respectively, 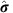 are the standard Pauli spin matrices and *U* the Coulomb scattering term, which was negligibly small for most calculations. In addition, spin–spin interactions of electrons originating and ending in the substrate were accounted for any spin, not only the one the tip is placed above. This matrix transition element results then in


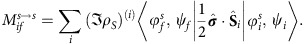


Here, ℑ*ρ*_*S*_ is the dimensionless coupling strength between the sample electrons and the localized spin with ℑ as the coupling energy and *ρ*_*S*_ the density of states in the sample close to the Fermi energy.

For all simulations we used an electron bandwidth *ω*_0_=20 mV and an effective temperature *T*_eff_=0.6 K, which best describes the effective linewidth of the excitations^22^. The anisotropies as well as the gyromagnetic factors of the three unperturbed impurities (Fe_hcp_, Fe_fcc_, Fe_hcp_H_2_) are known from earlier work^22^,^23^ and additionally have been extracted here by fitting spectroscopic data on individual impurities to the above model. The resulting parameters of the gyromagnetic factors *g* and uniaxial anisotropies are 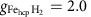, 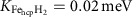, 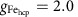, 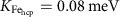, 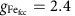, 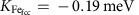. The coupling strengths ℑ*ρ*_*S*_, which also resulted from these fits, were ℑ*ρ*_*S*_=−0.12 for Fe_hcp_H_2_, ℑ*ρ*_*S*_=−0.02 for Fe_hcp_ and ℑ*ρ*_*S*_=−0.002 for Fe_fcc_. Thus, we are left with the coupling constants *J* and **D**, which we determine by calculating the conductance spectra for numerous settings and find the best match to the experimental data. To account for small drifts in the junction during measurements, we additionally allowed a small background slope and offset in d*I*/d*V*.

For the determination of the effective exchange field |*B*_eff_|, which mimics the splitting of the Kondo resonance, we fit the conductance spectra from the above model for a single magnetic atom, that is, without the interaction terms, to the data of the coupled Kondo atom, by adjusting **B** keeping *g*=2.

The same model has been used to calculate the spin-expectation values in out-of-plane 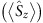, parallel 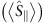 and perpendicular 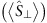 orientations (see assignment of directions in Fig. 1) as a function of **B**. These expectation values are proportional to the components of the magnetization of the impurities. Please note that, for the Kondo impurity Fe_hcp_H_2_, this is strictly only fully accurate as long as **B** is large enough to quench the Kondo screening^23^.

### First-principles calculations

The electronic and magnetic properties of the investigated dimers were obtained theoretically utilizing the full-potential KKR method^32^,^36^ that is based on density functional theory. Spin-orbit coupling was included self-consistently as it is an essential ingredient in the physics of the DMI.

In this type of investigation, the Pt(111) surface is first computed and then an embedding scheme based on the Green functions is employed to deposit the Fe adatoms on the substrate. The surface is simulated with a slab of 22 Pt layers augmented by two vacuum regions using the experimental lattice parameter of Pt (3.92 Å). To describe the adatoms, a real space cluster surrounding the adatoms is constructed. Pt is a 5d transition element that is initially non-magnetic but thanks to its high magnetic polarizability, it easily can develop a magnetic moment in the vicinity of Fe (ref. 22). This is also the case for the dimers. Thus, around each adatom, there is manifestation of a large induced spin-polarization cloud. Our previous calculations showed that, in order to reasonably describe the magnetic properties of an Fe adatom, the polarization of ∼60 Pt atoms neighbouring the impurity need to be taken into account^22^. Thus, for the dimers, twice this number of Pt atoms has to be included in our simulations. For the distances between the adatoms considered experimentally, the total magnetic moment of the dimers and substrate, including the spin and orbital components in the ferromagnetic state, reaches a large value of the order of ∼10μ_B_, 95% of which originates from the spin component. As soon as a hydrogen atom is attached to one of the adatoms, the total magnetic moment decreases by ∼0.8μ_B_. These results motivated our choice of attributing a spin of 5/2 for each Fe adatom in our model simulations.

Once the ferromagnetic state is obtained for all the considered dimers, a mapping procedure to a classical Heisenberg model is performed in order to extract the tensor of magnetic exchange interactions **J**^T^ (refs 37, 38). In this case, the Heisenberg Hamiltonian without the magnetic anisotropy energy and Zeeman term can be written as


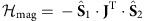


The nine elements of the tensor **J**^T^ can be extracted from *ab-initio* using infinitesimal rotations of the magnetic moments^37^,^38^ as:


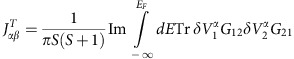


where *α*=(*x*, *y*, *z*) and *G*_12_ being the Green function connecting the atoms 1 and 2. *δV*^*α*^ represents the change of the adatom's potential upon infinitesimal rotation along the direction *α*. The prefactor *S*(*S*+1) takes care of the normalization followed in the model-based calculations presented in the main text. The trace of this tensor divided by 3 gives rise to the isotropic Heisenberg exchange interactions, whereas the antisymmetric part leads to the DMIs^37^,^38^. As can be seen from the previous equation, the presence of the Green function explains the oscillatory behaviour of *J* and **D** with respect to the mutual distance between the adatoms. Basically, the elements of the tensor **J**^**T**^ are described by electrons propagating from atom 1 to atom 2 and back while experiencing scattering at the magnetic part of both potentials.

Although, in principle, the whole tensor can be utilized to build a generalized Heisenberg Hamiltonian, we analysed its properties in order to reduce the quantities to be fitted experimentally to the most relevant ones and ended up with equation (1). Higher-order terms can be in principle added to the Hamiltonian in equation (1), for instance the so-called biquadratic term which is proportional to 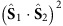. However, and as verified in our simulations, such a term is dramatically small for the distances considered experimentally. This is expected as the biquadratic contribution involves terms containing four Green functions that would describe the double propagation of electrons and their scattering at both adatoms when compared with the regular bilinear term *J*. Thus, the distance-dependent decay of the biquadratic interactions is expected to be stronger than the one characterizing *J*.

## Additional information

**How to cite this article:** Khajetoorians, A. A. *et al*. Tailoring the chiral magnetic interaction between two individual atoms. *Nat. Commun.* 7:10620 doi: 10.1038/ncomms10620 (2016).

## Supplementary Material

Supplementary Information Supplementary Figure 1-6, Supplementary Table 1 and Supplementary Notes 1-3

Supplementary Movie 1 The movie shows the magnetic field (*B_z_* given at the bottom of each panel) dependent evolution of the calculated expectation values 〈*Ŝ_z_*〉 (vertical axis) and 〈*Ŝ_‖_*〉 (horizontal axis) of Fe_hcp_H_2_ (left red line) and Fe_hcp_ (right red line) for three pairs of different separation *d* and signs of the perpendicular component of the Dzyaloshinskii-Moriya vector (*D*_⊥_) given on the top. It illustrates the resulting clockwise (left and right panels) and counterclockwise (middle panel) rotation of the magnetization.
