# Supplementary material for: Tailoring the chiral magnetic interaction between two individual atoms
Source: Nat Commun. 2016 Feb 23;7:10620. doi: 10.1038/ncomms10620 (PMC4766390; doi:10.1038/ncomms10620)
Supplement: Supplementary Information — Supplementary Figure 1-6, Supplementary Table 1 and Supplementary Notes 1-3 [file ncomms10620-s1.pdf]

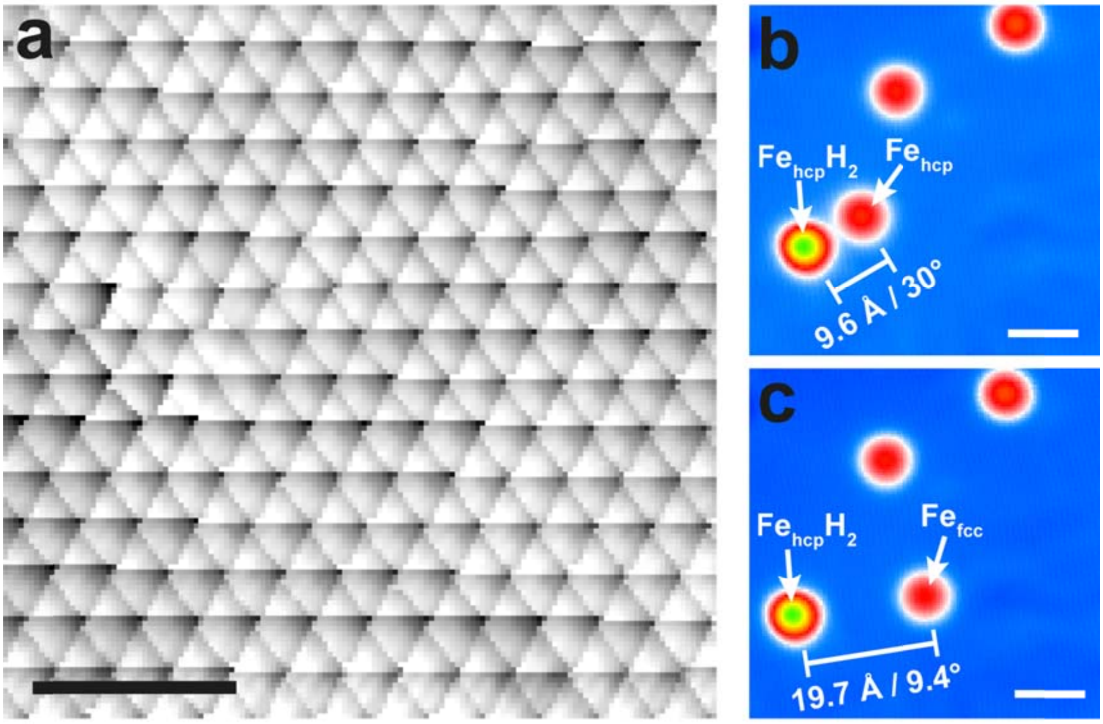

Supplementary Figure 1 | Determination of the atoms' positions on Pt(111)

Atomic manipulation image showing the fcc and hcp adsorption sites on the lattice of surface Pt atoms (**a**). Determination of the atoms' relative positions on the surface atomic lattice for two pairs with different distances and angles (**b,c**). All scale bars have a length of 1 nm.

Supplementary Note 1 | Results for additional pairs

In figure 4 of the main manuscript and in Supplementary Table 1, we show the experimentally determined parameters for Heisenberg and Dzyaloshinskii-Moriya interactions ($\boldsymbol{J}$ and $|\boldsymbol{D}_{\perp}|$, respectively), which have been extracted by fitting the simulation model to the experimental data. Due to limited space we only showed the

	EXPERIMENT		THEORY				
**d (in Å)**	**J (in μeV)**	**$D_{\perp}$ (in μeV)**	**J (in μeV)**	**$D_{\parallel}$ (in μeV)**	**$D_{\perp}$ (in μeV)**	**D_z (in μeV)**	**|D| (in μeV)**
6.98	10 ± 10	45 ± 5	-	-	-	-	-
7.34	-30 ± 10	20 ± 10	-39.3	-5.6	39.1	-29.2	49.1
8.31	-60 ± 15	50 ± 15	-33.7	0.0	-49.3	42.9	65.4
8.92	60 ± 15	50 ± 10	-	-	-	-	-
9.74	-10 ± 5	10 ± 5	-	-	-	-	-
10.0	-15 ± 10	25 ± 10	-18.2	-9.8	-9.8	1.2	13.9
11.1	-50 ± 10	30 ± 10	-18.9	0.0	21.9	3.4	22.2
12.5	10 ± 5	5 ± 5	-	-	-	-	-
13.9	0 ± 5	25 ± 5	-0.3	0.0	13.5	0.7	13.5
16.6	15 ± 5	20 ± 5	3.1	0.0	-4.4	4.5	6.2

Supplementary Table 1 | Comparative list of all extracted parameters

List of all experimentally determined parameters J and $|D_{\perp}|$ compared to the corresponding values from the first-principles calculations.

comparison between experiment and simulation for one distinct pair in the main manuscript. Supplementary Figures 2 to 4 show the corresponding comparisons between the experimental data and the fitted simulations for eight additional pairs.

Two distinguishing cases are illustrated in Supplementary Figure 2. For the $d = 8.92$ Å pair (Supplementary Figure 2a-l), we find that the Kondo peak splits nearly linearly with increasing B_z. Likewise, the Fe_{fcc} atom shows a linear increase in the excitation energy with increasing B_z. This indicates that both atoms easily align to the out of plane magnetic field, reminiscent of ferromagnetic coupling. As compared to our simulations, at this distance, J is the dominant term with no significant effect from **D**. Our simulations furthermore show that there is a distinct difference of the magnetic field response of the pair between $J > 0$ and $J < 0$, i.e. antiferromagnetic coupling would lead to a weaker response than seen experimentally.

For the $d = 13.9$ Å pair (Supplementary Figure 2m-x) there is no clear splitting of the Kondo peak for $B_z = 0$, which suggests an absence of magnetic coupling. However, by tracing the magnetic field dependence, we see that the evolution of the spectra on both, $Fe_{hcp}H_2$ and Fe_{hcp} differs from the uncoupled case. Only by considering a small DMI, namely $D_{\perp} \neq 0, J \approx 0$, is it possible to reproduce the experimental magnetic field dependence of the spectra for both atoms, especially for $Fe_{hcp}H_2$ at low magnetic

field. This indicates that the magnetic coupling can also be dominated solely by **D**. These examples show that the measured spectra are sensitive to the sign of J and to the relative strength between J and **D**.

Additional examples of the simulated spectra fitted to the experimental data are given in Supplementary Figures 3 and 4. Note, that during measurements in a magnetic field, we could not achieve building pairs with distances smaller than 7 Å. This is due to the $Fe_{hcp}H_2$ Kondo complex, which easily reacts with the tip in such close proximity.

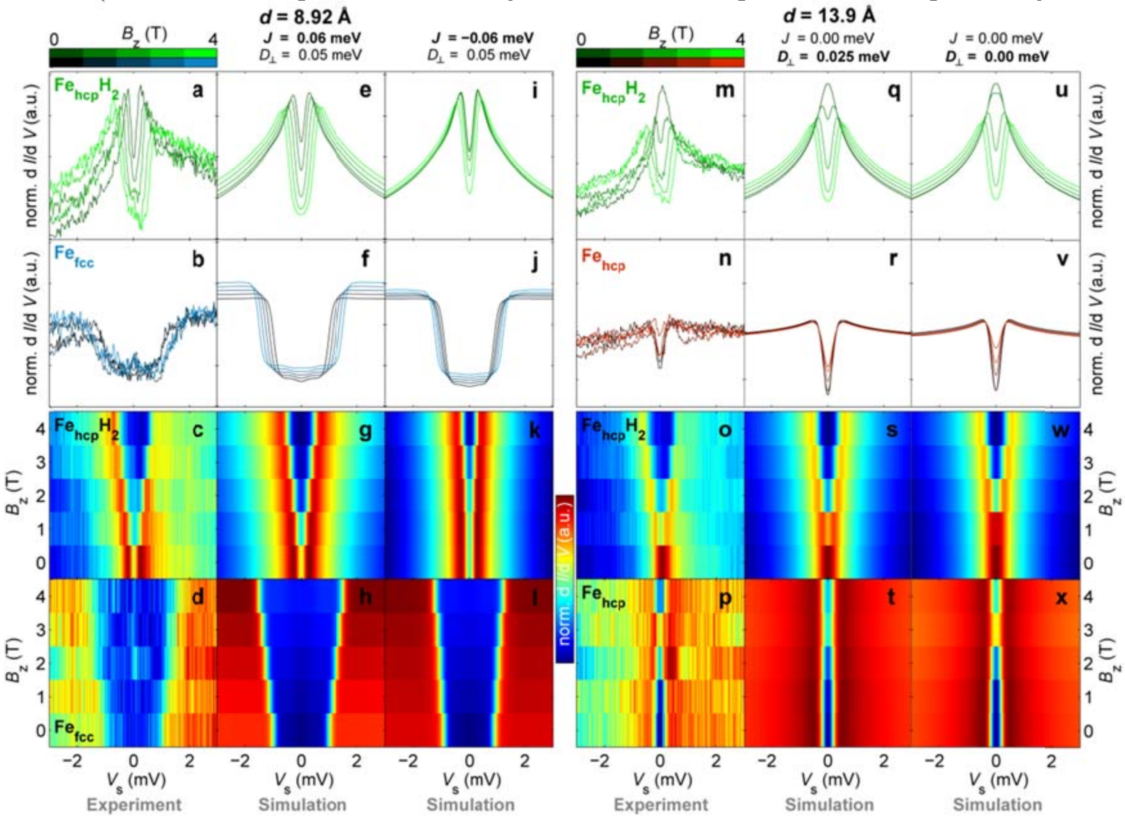

Supplementary Figure 2 | Proving the capabilities of the fitting procedure

Magnetic field dependent spectra and color intensity plots of the experimental ISTS data (**a-d, m-o**) and of the simulated data (**e-l** and **q-x**) of the $Fe_{hcp}H_2$ and Fe atoms in the $d = 8.92$ Å pair (**a-l**) and in the $d = 13.9$ Å pair (**m-x**). The values used for J and $|D_\perp|$ are given at the top above the simulations. The simulated spectra which best fit the experimental data are given in the middle panels.

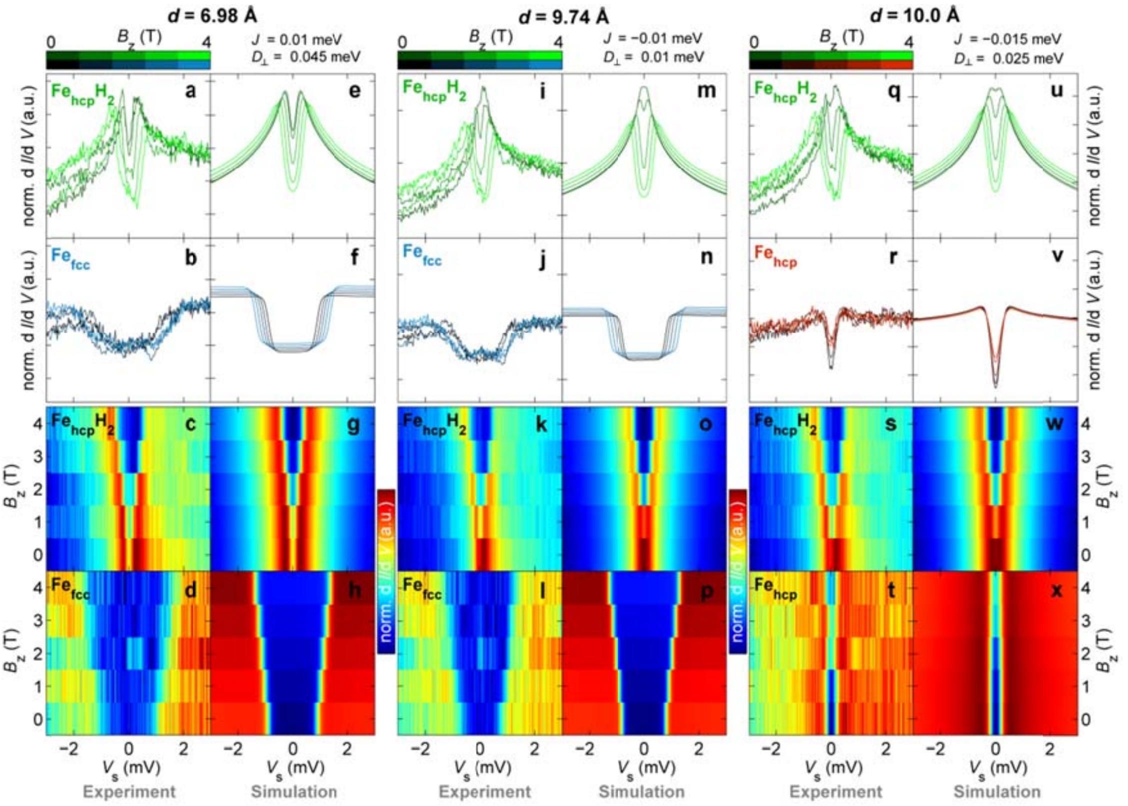

Supplementary Figure 3 | Fits for pairs having additional distances #1

Magnetic field dependent spectra and color intensity plots of the experimental ISTS data (**a-d**, **i-l**, **q-t**) and of the fitted simulated data (**e-h**, **m-p**, **u-x**) of the $Fe_{hcp}H_2$ and Fe atoms in three different pairs with the separation d given at the top. The fitted values of J and $|D_\perp|$ are indicated.

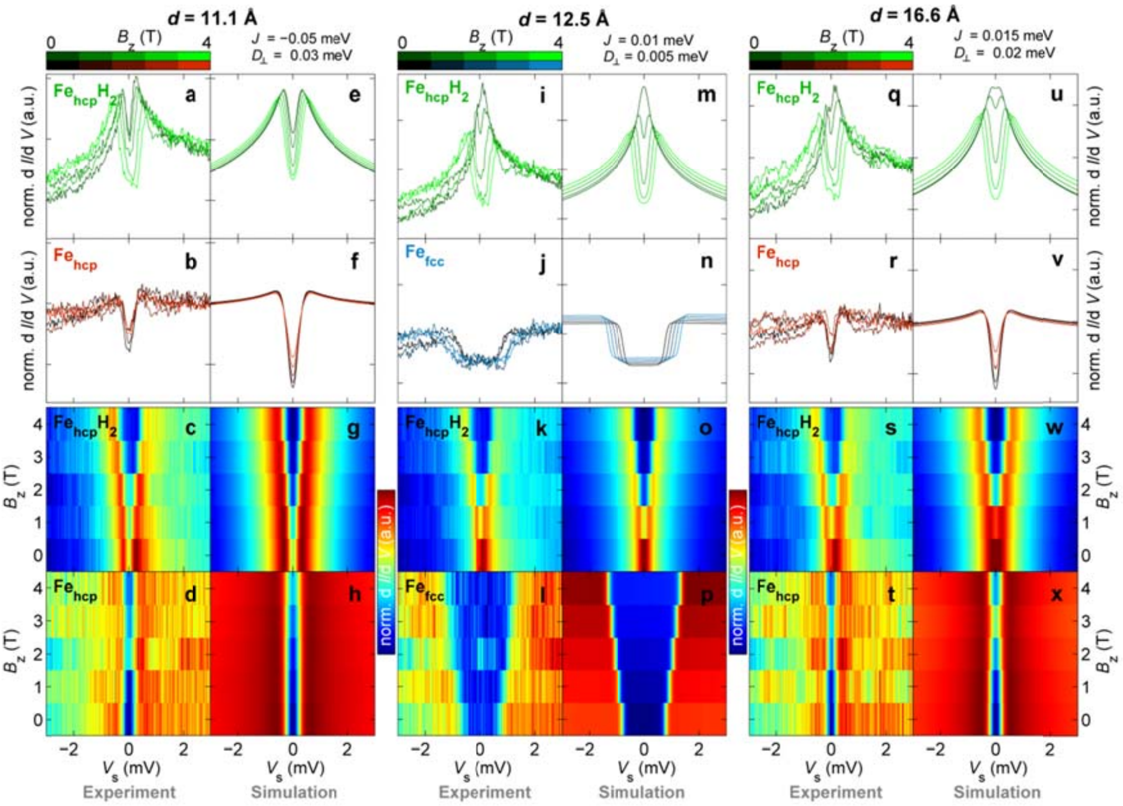

Supplementary Figure 4 | Fits for pairs having additional distances #2

Magnetic field dependent spectra and color intensity plots of the experimental ISTS data (**a-d**, **i-l**, **q-t**) and of the fitted simulated data (**e-h**, **m-p**, **u-x**) of the $Fe_{hcp}H_2$ and Fe atoms in three different pairs with the separation d given at the top. The fitted values of J and $|D_{\perp}|$ are indicated.

Supplementary Note 2 | Robustness of the fitting procedure

The robustness of our fits is exemplarily demonstrated in Supplementary Figure 5 for the $d = 13.9$ Å pair. The error margins of the fitted parameters J and $|D_\perp|$ have been estimated by variation of the simulated curves. Supplementary Figure 5 shows the situation for the best fit, for parameters which are still within the error margins but right at the border, and for a set of parameters outside of the error margins. For the latter case, the simulated spectra for Fe_{hcp}-H_2 already show a splitting at zero magnetic field, which does not accord with the data.

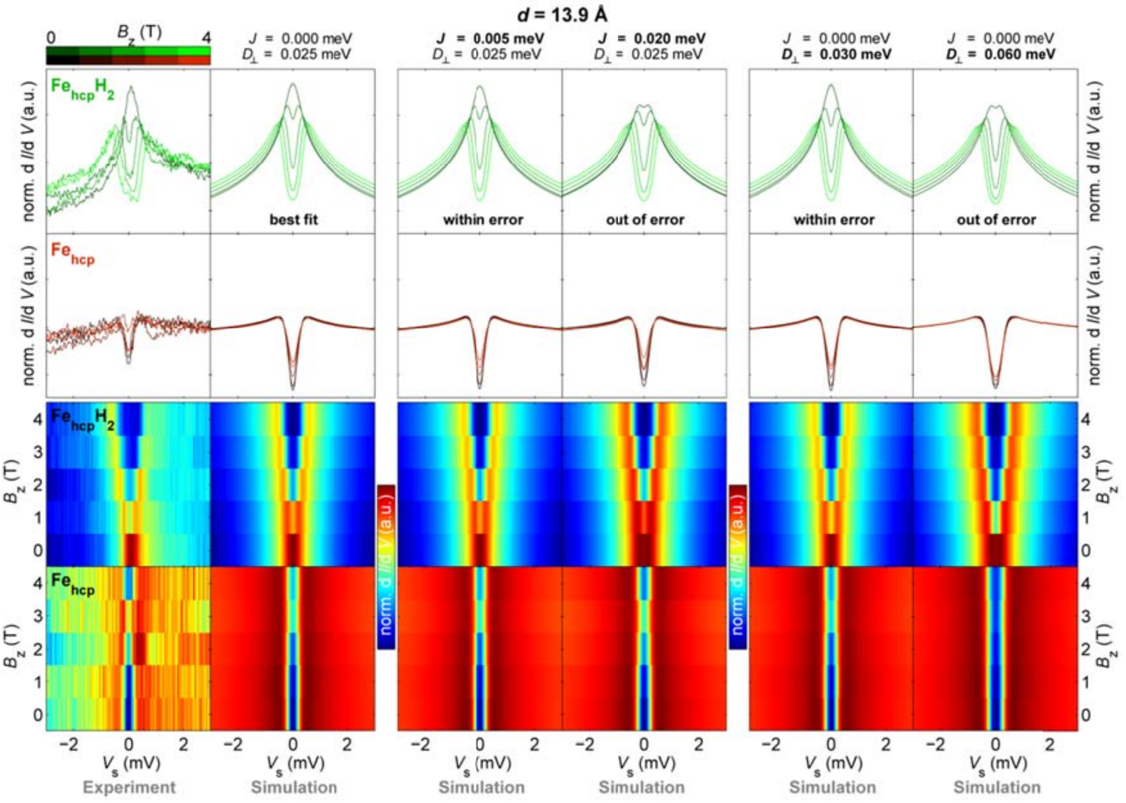

Supplementary Figure 5 | Demonstration of the robustness of the fits

Demonstration of the effect on simulated spectra by changing the fit parameters J and $|D_\perp|$ for the $d = 13.9$ Å pair. The simulated spectra are shown for values of the parameters corresponding to the best fit, to the border of the determined error, and to a value outside the error margin to show the clear differences.

Supplementary Note 3

To strengthen our approximation to only account for the in-plane components of **D** in our simulations, the out-of-plane component D_z of the calculation is plotted in Supplementary Figure 6. This component is negligible for most of the pairs and only has a significant value for distances $d < 9$ Å. The D_z contribution could possibly explain the larger error bar we obtain for the pair with 8.31 Å separation.

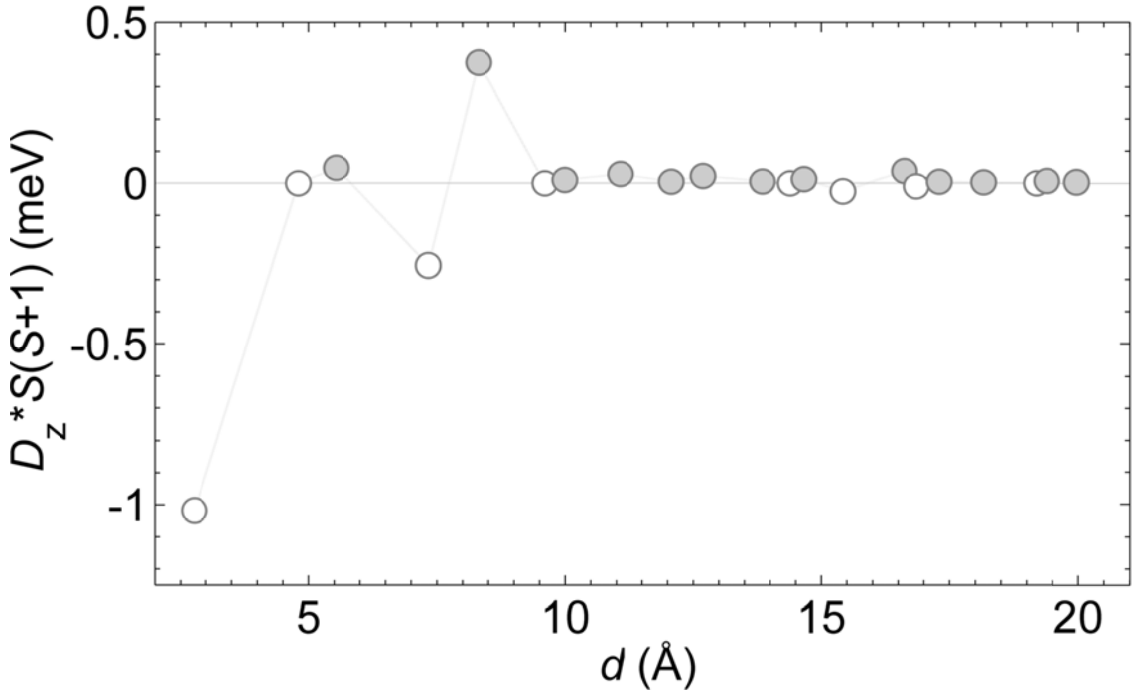

Supplementary Figure 6 | Calculated out-of plane component of D_z

Calculated out-of plane component D_z of the DM vector as a function of separarion d of the pair. Filled circles represent positive values, empty circles negative values, respectively.
